# A causal association between amyotrophic lateral sclerosis and atrial fibrillation: a two-sample Mendelian randomization study

**DOI:** 10.3389/fcvm.2024.1351495

**Published:** 2024-04-11

**Authors:** Yiren Yao, Hongyang Liu, Yang Gu, Xiaojin Xu, Xiwen Zhang

**Affiliations:** Department of Cardiology, The Affiliated Huaian No.1 People’s Hospital of Nanjing Medical University, Huai’an, China

**Keywords:** amyotrophic lateral sclerosis, atrial fibrillation, arrhythmia, causal relationship, Mendelian randomization

## Abstract

**Objectives:**

To look into the connection between amyotrophic lateral sclerosis (ALS) and atrial fibrillation (AF) using Mendelian randomization (MR).

**Methods:**

Two-sample MR was performed using genetic information from genome-wide association studies (GWAS). Genetic variants robustly associated with ALS and AF were used as instrumental variables. GWAS genetic data for ALS (*n* = 138,086, ncase = 27,205) and AF (*n* = 1,030,836, ncase = 60,620), publicly available from IEU Open. The specific MR protocols were Inverse variance-weighted (IVW), Simple mode, MR Egger, Weighted mode, and Weight median estimator (WME). Subsequently, the MR-Egger intercept and Cochran Q examine were used to evaluate instrumental variables (IVs)' heterogeneity and multiplicative effects (IVs). In addition, MR-PRESSO analysis was conducted to exclude any potential pleiotropy.

**Results:**

The IVW method demonstrated that ALS positively affected AF [OR: 1.062, 95% CI (1.004–1.122); *P *= 0.035]. Indeed, other MR methods were in accordance with the tendency of the IVW method (all OR > 1), and sensitivity testing verified the reliability of this MR result.

**Conclusions:**

This MR study proves a positive causal connection between ALS and atrial fibrillation. Further studies are warranted to elucidate the mechanisms linking ALS and AF.

## Introduction

Amyotrophic lateral sclerosis (ALS) is a neurodegenerative disease that causes the selective degeneration of upper and lower motor neurons in the motor cortex, brainstem, and spinal cord (1, 2). This causes progressive muscle weakness, atrophy, paralysis, and death, usually from respiratory failure, within 2–6 years of the onset of symptoms (1, 2). The average onset age is between 55 and 65 years, and the lifetime risk of ALS is approximately 1 in 300 (3). Most cases of ALS occur sporadically but about 5%–10% are familial, caused by inherited genetic mutations (3). The cause of sporadic ALS is still largely unknown. Nonetheless, suggested mechanisms include glutamate excitotoxicity and protein misfolding, oxidative damage, mitochondrial disease, and neuroinflammation (1, 4). As such, ALS remains an incurable disease with significant unmet medical needs.

Several cardiac arrhythmias can be observed among ALS. Such as supraventricular tachycardia, ventricular tachycardia, ventricular fibrillation, atrial fibrillation, long QT syndrome, and heart blocks (5). Epidemiological studies indicate that patients with ALS have higher rates of cardiovascular comorbidities, including coronary artery disease, stroke, and heart failure, compared to the general population (6). Studies have found that ALS can present with mild cardiovascular autonomic dysfunction in the early course of the disease, which increases the risk of arrhythmia and sudden death (7). Mandrioli et al. also discovered that cardiovascular diseases negatively associated with ALS included AF, heart failure, and hypertension, thus determining that cardiovascular disease may have a negative impact in the prognosis of ALS (8). Besides, Hoda et al. also found that AF is independent prognostic factors for the survival of ALS, and AF are independently associated with a shorter survival period of ALS (6).

AF is distinguished by rapid and disordered electrical activity in the atria, causing an irregular response from the heart (9). This leads to symptoms such as palpitations, fatigue, exercise intolerance, increased risk of heart failure, stroke, and death rates (9). In the general public, the lifetime risk of AF is about 25% (10).

While the association between ALS and AF has been documented, the nature and causality of this relationship remain unclear. One major limitation of previous observational studies is the inability to determine causality due to potential confounding and reverse causation biases. For instance, shared risk factors could predispose patients to ALS and AF. It is also plausible that AF could represent a consequence of ALS pathophysiology or that ALS may develop secondary to AF-related cerebral hypoperfusion and hypoxia (11). Therefore, elucidating the causal dynamics between ALS and AF has important implications. It may uncover novel etiological mechanisms and identify potential prognostic factors or therapeutic targets for ALS or AF.

Mendelian randomization (MR) has appeared as an effective technique to assess causal relationships between modifiable exposures and outcomes based on genetic variations as instrumental variables (IVs) (12). Given that different genetic variants are distributed at random during conception, MR is less susceptible to confounding (13, 14). Moreover, germline genetic variants precede disease onset. They are generally unaffected by the disease process itself, minimizing reverse causation. Two-sample MR utilizes summarizing genetic information from various genome-wide association studies (GWAS) of the exposure and outcome. This approach enhances statistical power by leveraging large GWAS datasets. MR techniques have been increasingly applied to investigate the causal relationships between various modifiable exposures and ALS or AF (15). For example, Xia et al. found a positive causal relationship between linoleic acid (LA) and ALS risk through MR analysis. At the same time, vitamin D was negatively correlated with ALS risk (16). Xia et al. also found a causal connection between increased telomere length and reduced hazard of ALS through MR analysis (17). Mohammadi-Shemirani et al. also found through MR analysis that Elevated Lipoprotein (a) is a potential causal mediator associated with increased risk of atrial fibrillation (15). However, MR studies examining the potential causal relationship between ALS and AF are lacking.

In this research, we used a two-sample MR analysis to look into the relationship between ALS and AF. utilizing GWAS data from large populations of European ancestry. Genetic instruments strongly associated with each trait were applied in bidirectional MR frameworks. Several sensitivity studies were carried out to assess and account for probable pleiotropy. Elucidating the possible causal relationship between ALS and AF will provide novel insights into disease mechanisms and may have important implications for patient prognosis, monitoring, and management.

## Methods and material

### Study design and data

**Figure 1:**
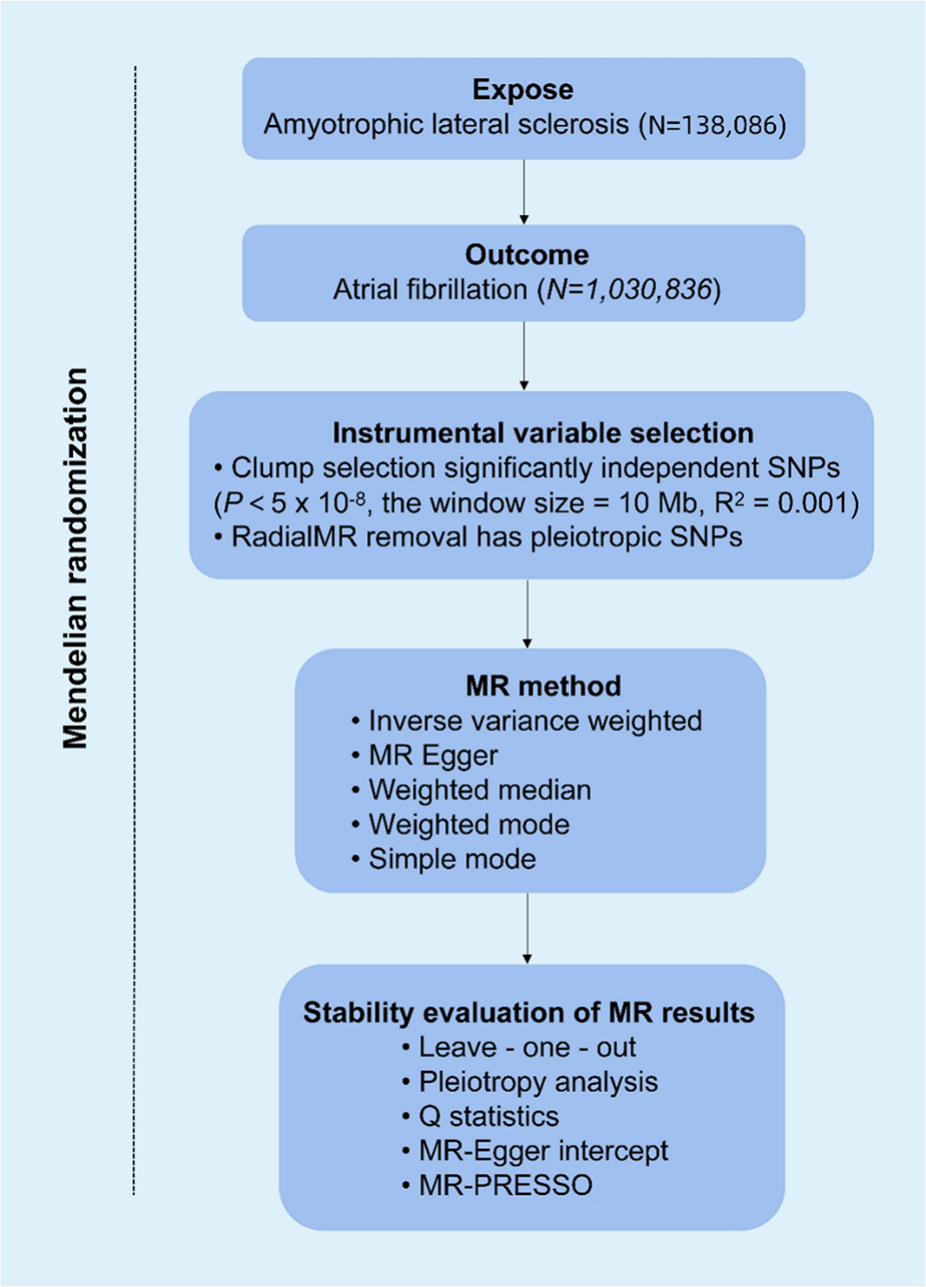
Overview of Mendelian randomization (MR) analysis of amyotrophic lateral sclerosis (ALS) and atrial fibrillation (AF).

Figure 1 shows the flowchart of the MR study. All of the genetic information used in this MR study was obtained from public sources, which are openly available on the IEU Open GWAS project, and specific ethical approvals were expressed in the original GWAS article. This study followed the three hypotheses of MR research: (i) genetic variation is strongly associated with risk factors; (ii) genetic variation is not associated with confounding factors; and (iii) genetic variation affects outcomes only through risk factors. GWAS data for ALS (ID: ebi-a-GCST90027164) and atrial fibrillation (ID: ebi-a-GCST006414) were found on the IEU Web site (https://gwas.mrcieu.ac.uk/), accessed on 2024-03-23. Table 1 provides details of the GWAS data sources. The pooled data for ALS included 138,086 participants (27,205 cases, 110,881 controls). The pooled data for AF consisted of 1,030,836 participants (60,620 cases, 970,216 controls). All participants were from the European population.

**Table 1:** Phenotype source and ALS and AF.

Phenotypes	PMID	ID	First author (Year)	Sample size	SNPs	N Cases	N Controls	Population
ALS	34873335	ebi-a-GCST90027164	van Rheenen W (2021)	138,086	10,427,126	27,205	110,881	European
AF	30061737	ebi-a-GCST006414	Nielsen JB (2018)	1,030,836	33,519,037	60,620	970,216	European

ALS, Amyotrophic lateral sclerosis. AF, Atrial fibrillation.

### Selection of instrumental variable (IVs)

In previous studies (18), significant Single nucleotide polymorphism (SNPs) were selected from the GWAS pooled data of ALS (selection criteria: *P *< 5 × 10^−8^, linkage disequilibrium *R*^2^ of 0.001, and linkage disequilibrium region width of 10,000 kb) to make sure each SNP was independent of each other and to omit the impact of genetic pleiotropy on the results. In addition, to quantify the level of gene variation, the *F*-statistic was calculated, and those SNPs with an *F*-statistic of 10 or higher were selected as the essential correlation criteria. The *F* statistic for specific individual SNPs was calculated as *F* = (*N *− 2)/*K* × *R*^2^/(1* *− *R*^2^), where *N* refers to the total number of samples contained in the exposure database, *K* refers to the total number of IVs and R^2^ is the proportion of variants explained by SNPs in the exposure database. *R*^2^ was calculated as *R*^2 ^= 2×EAF × (1 − EAF) × *β*^2^, where EAF stands for the effect allele frequency and *β* refers to the value of the allele's effect.

The above-filtered SNPs associated with ALS were then extracted from the GWAS pooled data of AF, and SNPs without suitable alternative loci were eliminated. Summarizing the information of the ALS and AF datasets, SNPs directly associated with AF were also eliminated (*P *< 5 × 10^−8^). Following the above steps, the remaining SNPs were finally used as IVs. A detailed description of the IVs used in this MR study is presented in Table 2.

**Table 2:** Characteristics of the genetic IVs at the genome-wide significance level (*P* < 5 × 10^−8^).

SNP	SE	Position	CHR	*β*	*P*	EA	OA	EAF	*R* ^2^	*F*
rs10463311	0.0126	15,04,10835	5	−0.0792	3.457 × 10^−10^	T	C	0.7474	0.002368464	27.31861908
rs113247976	0.0492	57,97,5700	12	0.3322	1.41906 × 10^−11^	T	C	0.0155	0.003368036	38.88695806
rs12608932	0.0121	17,75,2689	19	0.1247	8.76799 × 10^−25^	C	A	0.3472	0.007048923	81.68776744
rs17785991	0.0118	48,43,8761	20	0.0738	3.51301 × 10^−10^	A	T	0.3525	0.002486232	28.68037515
rs2045180	0.0118	31,07,8502	14	0.0922	5.69639 × 10^−15^	A	G	0.3353	0.003789231	43.76852901
rs2077492	0.0111	31,60,6392	6	0.0612	3.34603 × 10^−08^	A	G	0.4928	0.001872332	21.58533547
rs2453554	0.0126	27,56,1800	9	0.1723	2.56094 × 10^−42^	T	C	0.2482	0.011079104	128.9155212
rs517339	0.0112	17,23,54731	5	−0.0645	8.46408 × 10^−09^	C	T	0.3966	0.001991166	22.95805903
rs631312	0.012	39,50,8968	3	−0.0791	5.24204 × 10^−11^	A	G	0.7087	0.002583366	29.80378256
rs7303577	0.0174	64,87,4102	12	−0.098	1.75198 × 10^−08^	C	A	0.1116	0.001904386	21.95557712
rs75087725	0.0627	45,75,3117	21	0.4179	2.65216 × 10^−11^	A	C	0.0122	0.004209239	48.64045301
rs9275477	0.0207	32,67,2641	6	−0.1426	5.50174 × 10^−12^	C	A	0.0964	0.003542602	40.90964208

IVs, instrument variables.

### MR analysis approach

Several MR methods were utilized in this study to assess the causal relationship between ALS and AF. The primary analysis was carried out using the conventional Inverse variance weighted (IVW) method. The IVW method combines the estimates of each SNP using inverse-variance weighting to estimate the causal effect. IVW is a method used in MR to perform meta-analysis of the effects of multiple SNPs on multiple loci. The prerequisite for the application of IVW is that all SNPs are valid instrumental variables and independent of each other (13). To complement the IVW estimates, additional MR techniques such as MR-Egger regression, Weight median estimator (WME), Weighted mode and Simple mode were also carried out. These approaches can provide more robust causal inferences under varying scenarios. MR-Egger regression has the ability to identify and take into account heterogeneity and unbalanced pleiotropy (12). The WME approach gives consistent effect estimates in the event that at least fifty percent of the genetic variants are valid instruments (19). Simple mode is the simplest MR analysis strategy. Its calculation is straightforward and easy to use, and even if it contains a small number of weak instrumental variables, it can provide a relatively accurate estimation of causal effects (13).

### Sensitivity evaluates

In order to evaluate the dependability of the MR estimates, a number of different sensitivity evaluates were carried out. The possible horizontal pleiotropy of the instruments was taken into account with MR-Egger regression. Leave-one-out cross-validation was conducted by iteratively removing each SNP from the instrument sets. Between-SNP heterogeneity was quantified using Cochran's Q statistic. In addition, funnel plots visually depicting SNP-specific effects against instrument strength were assessed to check for directional pleiotropy.

### Statistical analysis

The R software's TwoSampleMR package was used to carry out the MR analyses (for more information, visit http://www.R-project.org). In each of the analyses, statistical significance was defined as a *P*-value that was lower than 0.05.

## Results

### ALS has a positive causal relationship with AF

**Figure 2:**
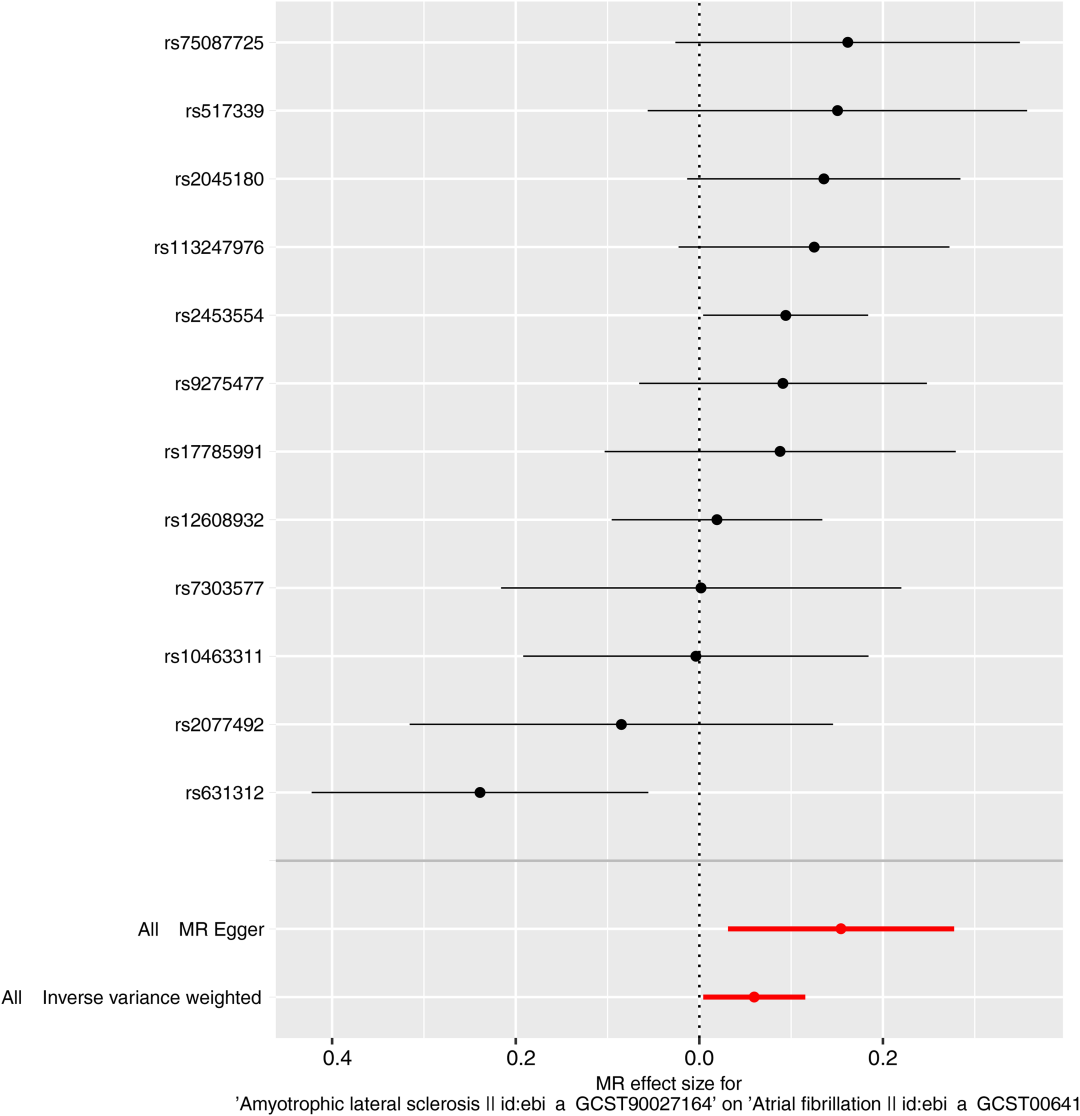
Forest plot of MR analysis of ALS and AF.

**Figure 3:**
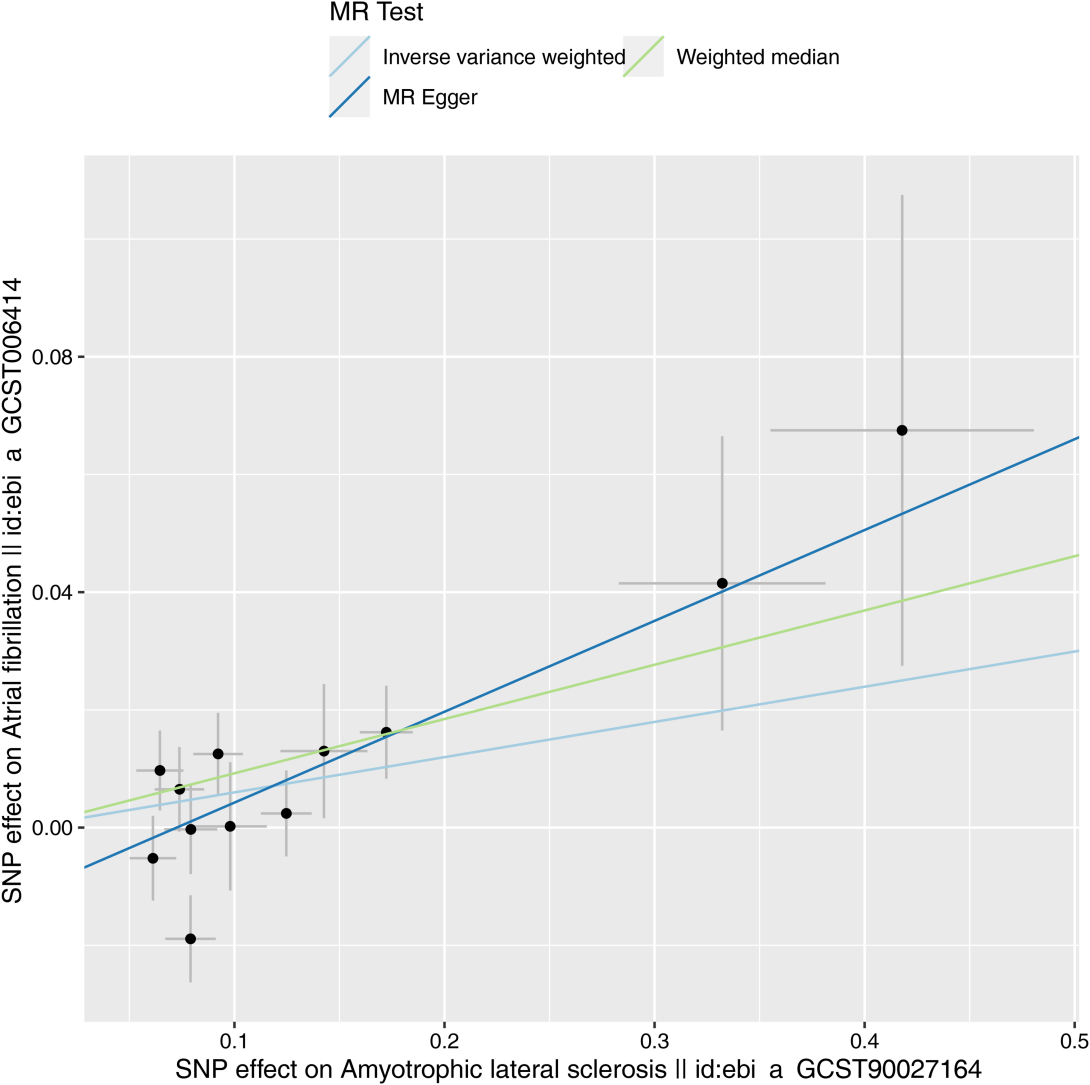
Scatter plot of MR analysis of ALS and AF.

Characteristics of the genetic IVs at the genome-wide significance level are presented in Table 2, including the SNP ID, effect allele, reference allele, SNP position, Chr, Beta, Se and P. Statistically, most of the *F*-statistics for the IVs were more than 10, reducing the likelihood that this study had weak IVs. Five MR methods were used in this study to examine the causal outcomes, with the IVW approach as the primary method to determine causality. As shown in Table 3, the IVW method demonstrated a positive causal relation of ALS on AF [OR: 1.062, 95% CI (1.004–1.122); *P *= 0.035]. Similarly, the Weighted median also demonstrated a positive causal relation of ALS on AF [OR: 1.097, 95% CI (1.030–1.167); *P *= 0.004]. The other three MR methods, such as MR Egger [OR: 1.167, 95% CI (1.032–1.320); *P *= 0.034], Simple mode [OR: 1.116, 95% CI (1.023–1.218); *P *= 0.032], and Weighted mode [OR: 1.102, 95% CI (1.026–1.183); *P *= 0.022], although they cannot separately display a causal relationship between ALS and AF, the OR direction remains consistent with IVW, which also supports the results of this IVW (Table 3, Figure 2). Specifically, the effect of each SNP in the MR analysis is shown in Figure 3. The results consistently showed a positive causal effect of ALS on AF risk.

**Table 3:** The causal relationship between ALS and AF through MR analysis.

MR method	*β*	OR (95% CI)	*P*
MR Egger	0.154	1.167 (1.032–1.320)	0.034
Weighted median	0.092	1.097 (1.030–1.167)	0.004
IVW	0.060	1.062 (1.004–1.122)	0.035
Simple mode	0.110	1.116 (1.023–1.218)	0.032
Weighted mode	0.097	1.102 (1.026–1.183)	0.022

Inverse variance weighted.

### Sensitivity analyses

**Figure 4:**
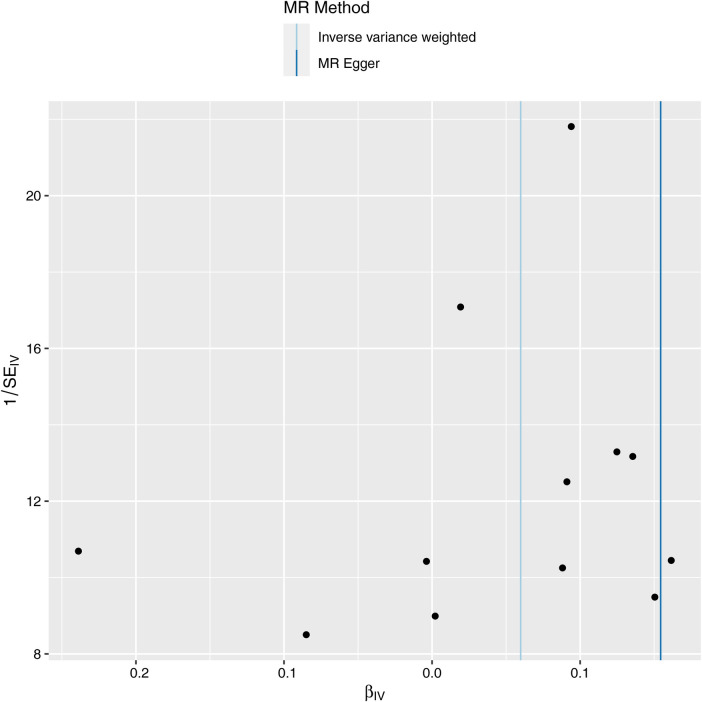
Funnel plot of MR analysis of ALS and AF.

**Figure 5:**
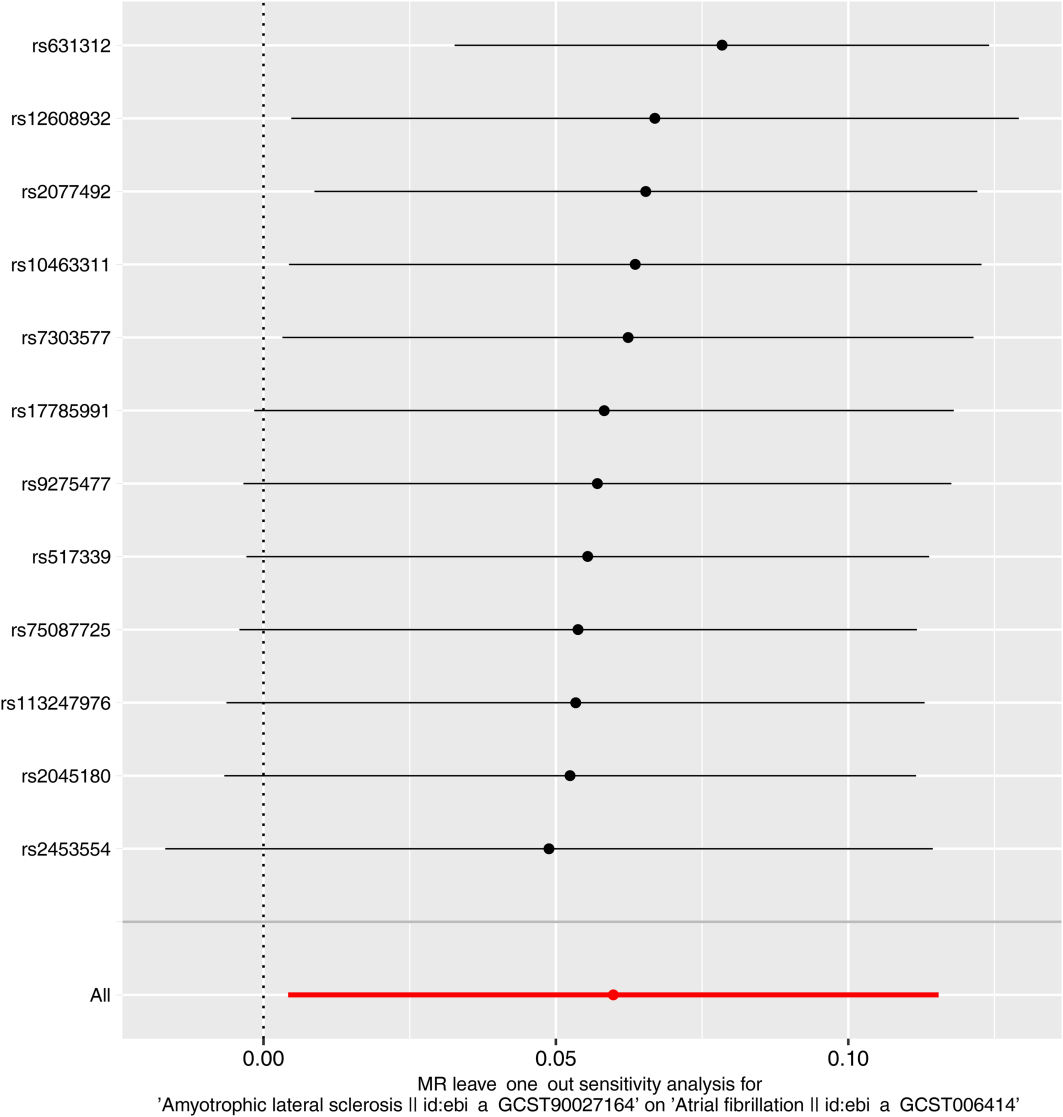
The leave-one-out plots for MR analysis of ALS and AF.

Next, we evaluated the differences in IVs, and the funnel plot showed that all IVs were roughly evenly distributed without significant deviation (Figure 4). The leave-one-out examination of the remaining method indicates that the relation between ALS and AF does not stem from a single SNP (Figure 5). The MR-Egger intercept revealed no substantial horizontal pleiotropy for these IVs (*P *= 0.128). In addition, the Cochran *Q* test results based on the IVW method also showed no heterogeneity in IVs (*P *= 0.099). Finally, the MR-PRESSO analysis also showed no potential pleiotropic outliers (*P *= 0.129).

In summary, the ALS genetic instruments demonstrated indication for a potential positive causal effect on AF risk.

## Discussion

This two-sample MR study supports a causal association between ALS and an increased risk of AF. The primary IVW analysis demonstrated a statistically significant positive causal effect of ALS on AF risk, with an odds ratio (OR) of 1.062 (95% CI 1.004–1.122, *P *= 0.035). This finding was corroborated by consistent directionality of effect estimates across several complementary MR approaches, including the WME, Simple mode, MR Egger, and Weighted mode models. Multiple sensitivity analyses showed no evidence of significant horizontal pleiotropy or heterogeneity among the genetic instruments. These results indicate that ALS may causally contribute to increased susceptibility to AF.

The potential mechanisms uncovering this association remain fully elucidated but may involve both central and peripheral pathophysiological processes related to ALS that predispose to cardiac arrhythmia (6). ALS is defined by the progressive degeneration and death of upper and lower motor neurons in the motor cortex or brainstem, which results in muscular atrophy and weakness (1). Autonomic nervous system dysfunction manifested as abnormal heart rate variability has been documented in ALS patients, even at early stages, indicative of cardiac autonomic imbalance (6). This autonomic neuropathy likely contributes to the increased risk of cardiac arrhythmias and sudden death observed in ALS (20, 21). At the tissue level, neurodegenerative changes in brain regions involved in central autonomic control, such as the hypothalamus, medulla, and intermediolateral cell columns of the spinal cord, may disrupt standard cardiovagal modulation and promote sympathoexcitation (22). Loss of inhibitory cortical inputs to the brainstem and spinal cord autonomic nuclei could further exacerbate autonomic dysfunction in ALS (6). Peripheral denervation of postganglionic sympathetic neurons innervating the heart may also directly alter electrophysiological properties and automaticity of cardiomyocytes, providing substrate for AF (23).

Furthermore, oxidative stress and mitochondrial dysfunction, key pathophysiological processes in ALS pathogenesis, likely also contribute to cardiac dysregulation and arrhythmogenesis (4, 24). Reactive oxygen species generation and antioxidant depletion intrinsically alter calcium handling, membrane excitability, and conduction in cardiomyocytes, facilitating triggered activity and re-entry (25). Impaired mitochondrial energetics and dysfunctional quality control further promote oxidative damage and protein aggregation in conduction system tissue (26, 27). Besides, neuroinflammation is another salient feature of ALS mediated by reactive astrocytes, microglia, and cytokines such as TGF-β, TNF-α, and IL-6 (28). Chronic inflammation is linked with AF susceptibility and commonly observed in atrial biopsy specimens from AF patients (29). Pro-inflammatory cytokines may contribute to fibrotic remodeling of atrial tissue as well as direct effects on ion channels and calcium handling proteins that influence arrhythmogenesis (29). The relationship between systemic inflammation and AF was further supported by Liu et al., showing IL-6 and TNF-α levels had causal effects on increased AF risk (29). Hence, neuroinflammation in ALS could potentially mediate AF via circulating cytokines.

Additional pathways by which ALS may lead to AF include respiratory compromise and acid-base abnormalities from progressive respiratory muscle weakness (30). Hypoxemia, hypercapnia, and acidosis can directly provoke arrhythmias and may exacerbate underlying cardiovascular dysfunction in ALS patients (31).

Our study has several strengths supporting the validity of its findings. MR circumvents biases from confounding and reverse causation inherent to conventional epidemiological studies by utilizing genetic instruments rather than directly measured phenotypes. Two-sample MR leverages massive GWAS datasets, enhancing statistical power to detect causal effects. The SNPs applied as instruments for ALS were selected based on stringent criteria and passed all prerequisite assumptions. The MR results were robust across several complementary analysis methods, and sensitivity analyses showed no significant issues with pleiotropy or heterogeneity.

In brief, this two-sample MR study provides evidence that ALS may play a causal role in increasing susceptibility to AF, potentially mediated through autonomic dysfunction, oxidative stress, neuroinflammation, respiratory compromise, and medication effects. The findings add to prior observational data associating ALS with more significant cardiovascular morbidity and earlier mortality. This relationship appears biologically plausible given pathophysiological processes in ALS predisposing to cardiac dysregulation and arrhythmogenesis. AF may serve as a prognostic factor in ALS and portend more significant risks of complications that could hasten functional decline. Clinicians may need heightened vigilance for cardiac rhythm abnormalities when evaluating and managing ALS patients. Future research should continue exploring this association's underlying mechanisms and translational implications. Confirmation across other large cohorts and ancestries is warranted. Exploring potential druggable targets influencing both diseases could yield therapeutic advances. This study provides novel insights into the causal relationships between neurodegenerative and cardiovascular disorders.

However, some limitations should be acknowledged. Given the rarity of ALS, there is currently a lack of observational studies confirming a higher incidence of AF in ALS patients. Therefore, the relevance of assessing the impact of ALS on AF risk seems limited. Despite large sample sizes overall, the number of eligible genetic instruments for ALS was modest, restricting analytical power. The populations studied were of European ancestry, limiting generalizability to other ethnicities. Also, We cannot exclude the possibility of residual pleiotropy from unmeasured traits influencing the results. MR estimates represent the lifetime effects of genetically determined exposures, whereas acquired factors modifying disease risk may further mediate observed associations. As summary data were used, exploring gene-environment or gene-gene interactions was impossible. Finally, the specific biological mechanisms linking ALS and AF remain speculative based on our analysis. Further studies must elucidate the pathophysiological pathways involved and confirm reproducibility in other populations.

## Conclusion

In conclusion, this two-sample MR study shows that ALS causally increases AF risk, though the biological mechanism requires further research. More studies are needed to confirm this association and uncover the underlying pathways.

## Data Availability

Publicly available datasets were analyzed in this study. This data can be found here: https://gwas.mrcieu.ac.uk/, ebi-a-GCST005647, ebi-a-GCST006414.
